# Perceptions of Orthopaedic Sports Medicine Surgeons About Medical Cannabidiol Use: A Survey Study

**DOI:** 10.7759/cureus.51759

**Published:** 2024-01-06

**Authors:** Ajith Malige, Alexandra Boyd, Orr Limpisvasti

**Affiliations:** 1 Orthopaedic Surgery, Cedars-Sinai Kerlan-Jobe Institute, Los Angeles, USA

**Keywords:** medical education, pain regimen, cbd awareness, opioid-sparing analgesia, orthopedic sports medicine, orthopaedic surgery, cannabidiol (cbd)

## Abstract

Introduction: Multiple studies exist identifying cannabidiol (CBD) as an effective part of an orthopaedic patient’s pain regimen; however, there is a paucity of studies elucidating orthopaedic surgeons' perception of the use and prescription of CBD in the medical setting. This study surveys orthopaedic sports medicine surgeons about their previous education on and current perceptions and usage of CBD in their medical practice.

Methods: Between April 2023 and July 2023, orthopaedic sports medicine surgeons from across the country were surveyed. This survey was designed in hopes of identifying physician perceptions and current use of CBD as well as their previous education and training on its use.

Results: Overall, 75 orthopaedic surgeons responded. More than three-fourths of responders had not received formal education on medical CBD use, nor did they have partners or colleagues who used CBD in their practice. More than half of all surgeons believed that there is a stigma associated with CBD use. A higher proportion of surgeons from CBD legal states recommended CBD to help patients control their pain (53.7% vs. 37.5%). Less than 15% of responders believed that CBD can adversely affect surgical outcomes. Finally, four-fifths of all responders believed that CBD is easy to legally access and affordable to buy by patients who desire it.

Discussion: The relative novelty of CBD inclusion in medicine has led to a lack of early education and overall experience with its use among orthopaedic sports medicine surgeons. Still, surgeons believe that CBD is a safe and effective option to control pain. As surgeons continue to gain more familiarity and trust with CBD’s medical uses over time, it has the potential to be a mainstay in orthopaedic multimodal pain regimens.

## Introduction

The legalization of marijuana or *Cannabis sativa* and *Cannabis indica* across the United States has led to its recent incorporation into healthcare. Marijuana has two main components, tetrahydrocannabinol (THC) and cannabidiol (CBD), which can both be isolated and applied. Via cannabinoid receptors both in the brain and peripheral tissue, THC increases dopamine and produces psychotropic effects [1], leading to both recreational and entheogenic utilization. In contrast, CBD has been shown to act on opioid receptors [2], potentially providing analgesia without the psychoactive effects. This has led to its utilization across various medical fields as part of a multimodal pain regimen aimed at better controlling pain and decreasing opioid use in the face of the opioid pandemic [3].

Due to multiple factors, including recent legalization and publicity [4], marijuana use has been increasing over the past 20 years [5]. Its potential to help with nausea, vomiting, and other symptoms with relative safety has increased its medical uses over the years [6]. This has even led to the classification of Epidiolex®, a CBD product as a generally recognized as safe (GRAS) product for medical use by the United States Food and Drug Administration (FDA). However, CBD’s analgesic effects have garnered the most attention and are its most common reason for usage, with both oral and topical forms commonly utilized. Medical marijuana is legal in 37 states, three territories, and the District of Columbia [7], making medical CBD use an evolving and important pain control option to consider. Orthopaedic patient pain has often been cited as one of the hardest to control [8], making CBD a natural target to utilize in hopes of better closing the gap between patient-perceived pain levels and overall pain control [9].

Multiple studies exist identifying CBD as an effective part of an orthopaedic patient’s pain regimen, both in treating pain secondary to non-operative conditions [10] and postoperative pain [11]; however, there is a paucity of studies elucidating orthopaedic surgeons' perceptions about the use and prescription of CBD in the medical setting. While 88% of trauma surgeons who were surveyed as part of the Orthopaedic Trauma Association (OTA) did not believe they were knowledgeable about the mechanism of CBD, 73% believed it could help control postoperative pain [12]. Unfortunately, no similar study exists to elucidate physician perceptions on CBD use among orthopaedic sports medicine surgeons. Therefore, this study seeks to survey orthopaedic sports medicine surgeons to identify previous education on and current perceptions and usage of CBD in their medical practice.

## Materials and methods

Approval for this cross-sectional survey study was given by the Cedars-Sinai Institutional Review Board (approval number: STUDY00002418). From April 2023 to July 2023, all orthopaedic sports medicine surgeons who were part of the American Orthopaedic Society of Sports Medicine (AOSSM) as well as surgeons from a large sports medicine fellowship alumni network (with multiple surgeons in every geographic region) were surveyed. All surveys that were completed were included in the study. This survey was designed in hopes of identifying physician perceptions and current use of CBD as well as their previous education and training on its use (See Appendices). This survey was constructed utilizing previously published surveys exploring patient CBD use [13-15]. The survey was distributed online utilizing REDCap web application (Vanderbilt Institute for Clinical and Translational Research, Nashville, Tennessee, United States).

The first section of the survey asked questions about surgeon demographics, including practice location and total years in practice. The next section asked whether surgeons were educated on CBD use in their medical training and if they have experience with its use in practice (by them or their colleagues). Surgeons are also asked if they believe there is a stigma associated with the medical use of CBD. Finally, surgeons are asked if they believe CBD use is effective in controlling pain and other pathologies (sleep and anxiety), whether certain forms of CBD are more effective than others, and whether CBD use can negatively affect patient outcomes. Data was recorded using REDCap [16,17]. Survey answers were analyzed utilizing descriptive statistics using IBM SPSS Statistics for Windows, Version 23.0 (Released 2015; IBM Corp., Armonk, New York, United States). For all analyses, statistical significance was set at p < 0.05.

## Results

Overall, 75 orthopaedic surgeons responded to our survey. Sixty-seven (89.3%) of these surgeons were from states where the medical use of CBD is legal. Most surgeons were either five or fewer years into practice and in private practice. There were also 13 surgeons who believed the medical use of CBD in their state was not legal when it truly was (Table 1).

**Table 1:** Demographics of Surveyed Population Percentages displayed as proportion of total patients in each cohort (orthopaedic surgeons in states where medical CBD use is legal versus states where it is not legal); Data presented as N (%) CBD=cannabidiol

		Medical CBD Legal States	Medical CBD Non-Legal States	Total
Years in Practice	<5 years	24 (35.8%)	3 (37.5%)	27 (36.0%)
	6-10 years	8 (11.9%)	1 (12.5%)	9 (12.0%)
	11-20 years	15 (22.4%)	1 (12.5%)	16 (21.3)
	21+ years	20 (29.9%)	3 (37.5%)	23 (30.7%)
Type of Practice	Academic	11 (16.4%)	4 (50.0%)	15 (20.0%)
	Hospital Employed	20 (29.9%)	0 (0.0%)	20 (26.7%)
	Private Practice	36 (53.7%)	4 (50.0%)	40 (53.3%)
Belief in State CBD Legality	Yes	54 (80.6%)	0 (0.0%)	54 (72.0%)
	No	13 (19.4%)	8 (100.0%)	21 (28.0%)
Total		67	8	75

More than 80% of orthopaedic surgeons from CBD legal states have not received formal education about medical CBD use, nor have they ever seen CBD used in their training. In CBD non-legal states, 62.5% (n=5) of surgeons have not received formal education about medical CBD use, while 75.0% (n=6) of surgeons have never seen CBD used in their training. About three-fourths of responders in both CBD legal and non-legal states did not have partners or colleagues that used CBD in their practice either. Finally, 58.2% (n=39) of surgeons from CBD legal states and 50.0% (n=4) of surgeons from CBD non-legal states believed that there is a stigma associated with CBD use (Table 2).

**Table 2:** Responder Answers to Questions about Education on CBD Use, Experience Using CBD, and Stigma Attached to CBD Percentages displayed as proportion of total patients in each cohort (orthopaedic surgeons in states where medical CBD use is legal versus states where it is not legal); Data presented as N (%) CBD=cannabidiol

		Medical CBD Legal States	Medical CBD Non-Legal States	Total
Have you received formal education about CBD and its medical uses?	Yes	6 (9.0%)	3 (37.5%)	9 (12.0%)
	No	61 (91.0%)	5 (62.5%)	66 (88.0%)
Did you ever see CBD used in your training?	Yes	11 (16.4%)	2 (25.0%)	13 (17.3%)
	No	56 (83.6%)	6 (75.0%)	62 (82.7%)
Do your partners or colleagues use CBD in their practice for pain control?	Yes	14 (20.9%)	2 (25.0%)	16 (21.3%)
	No	53 (79.1%)	6 (75.0%)	59 (78.7%)
Do you believe there is a stigma for using CBD (as a provider or for patients)?	Yes	39 (58.2%)	4 (50.0%)	43 (57.3%)
	No	28 (41.8%)	4 (50.0%)	32 (42.7%)
Total		67	8	75

**Figure 1:**
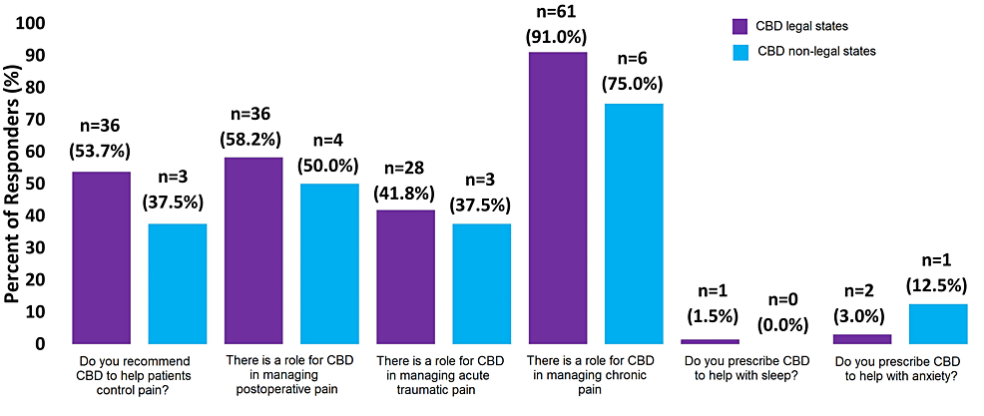
Survey Questions About Medical CBD Prescriptions CBD=Cannabidiol Percentage of respondents indicates respondents who have said “Yes” to these questions

A higher proportion of surgeons from CBD legal states recommend CBD to help patients control their pain (53.7% vs. 37.5%). Slightly more surgeons from these states also thought there is a role for CBD in managing postoperative pain (58.2% vs. 50.0%), acute traumatic pain (41.8% vs. 37.5%), and chronic pain (91.0% vs. 75.0%). Additionally, very few surgeons prescribed CBD to help with sleep (1.5% vs. 0.0%) or anxiety (3.0% vs. 12.5%) (Figure 1).

**Figure 2:**
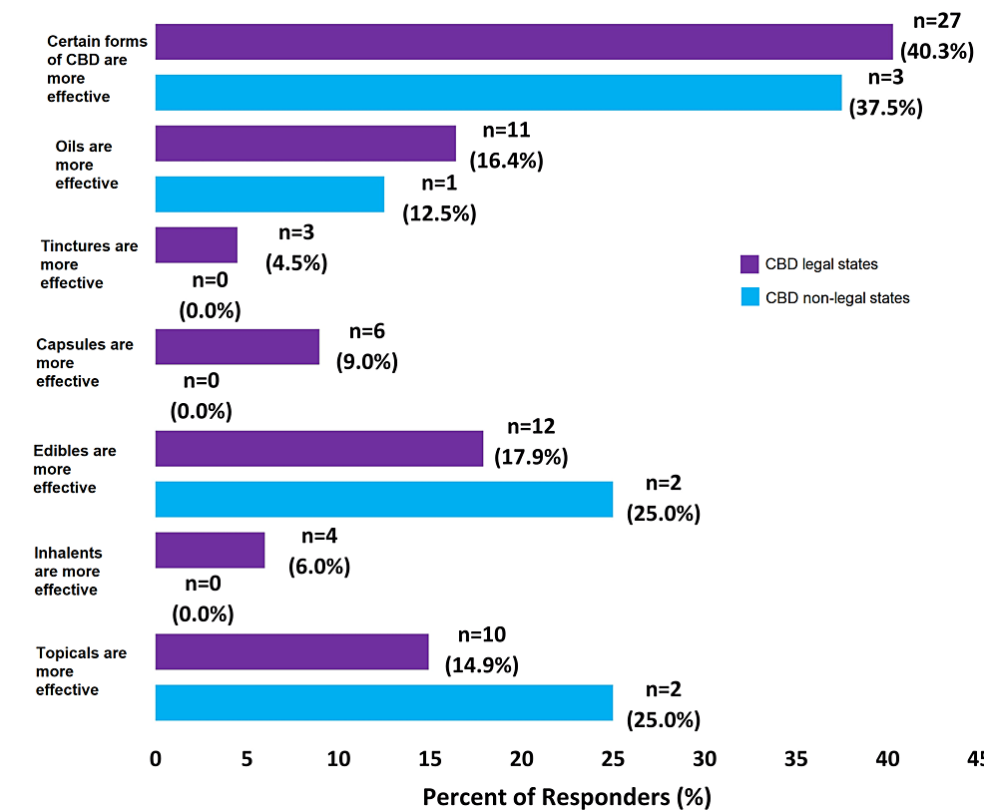
Survey Questions About the Effectiveness of Different Forms of CBD CBD=Cannabidiol Percentage of respondents indicates respondents who have said “Yes” to these questions

**Figure 3:**
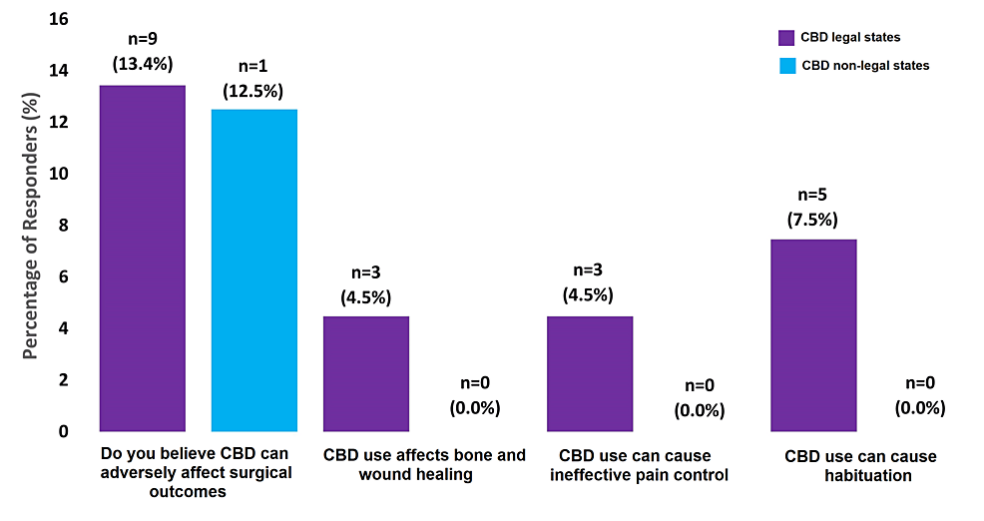
Survey Questions About CBD Side Effects CBD=Cannabidiol Percentage of respondents indicates respondents who have said “Yes” to these questions

While 40.3% (n=27) of responders in CBD legal states and 37.5% (n=3) of responders in CBD non-legal states believed that certain forms of CBD are more effective than others, there was no consensus among surgeons about which forms are more effective (Figure 2). Only 13.4% (n=9) of responders in CBD legal states and 12.5% (n=1) of responders in CBD non-legal states believed that CBD can adversely affect surgical outcomes, with less than 10% of responders noting that they believe CBD affects bone and wound healing, that it can cause ineffective pain control, and that it can cause habituation (Figure 3). Finally, a high percentage of responders in both CBD legal and non-legal states believed that CBD was easy to legally access for patients who desire to (83.6% vs. 87.5%) and is affordable to buy by patients who desire to (79.1% vs. 87.5%).

## Discussion

As CBD use in society increases in conjunction with increased legality laws, physicians must continue to explore the role of medical CBD in patient care. Current laws designate CBD as either conditionally legal or fully legal in all 50 states, allowing for increased CBD production as well as access for physicians and patients. CBD presents an intriguing option as part of a multimodal pain regimen with the goal of decreasing narcotic use in the face of the opioid pandemic [18]. However, physician education about and experience with CBD use is lacking, mostly due to the relative novelty of CBD legality. Still, responding surgeons believed both that there is a role for CBD in controlling chronic pain and that CBD use is relatively safe.

CBD education should start in the early stages of provider training in order to broaden understanding and experience over time. However, given the relatively new changes in CBD legality laws across the country [19], it has still not reliably become a part of medical education [20,21]. Responding physicians to our survey also had limited education and experience with CBD, both in training and in practice, a fact echoed in Chin et al.’s survey querying OTA members [12]. However, as CBD use continues to increase, hopefully so will early trainee education as well as overall physician comfort. This has the potential to decrease the overall stigma associated with its use, both among providers and patients.

Most surgeons did believe that CBD does have a place in treating pain, specifically chronic pain. This was also echoed by OTA members [12]. CBD’s efficacy in treating acute and chronic pain has been well established in the literature [11]. CBD does represent an effective alternative to narcotic medication to treat all types of pain, and all physicians should consider CBD in pain regimens in the right patient. In addition, only a few surgeons utilized CBD to treat sleep or anxiety, a use that has been well studied in the literature [22,23]. As CBD use becomes more prevalent in the face of increased state legality laws, we may see an increase in CBD use in various fields and for various uses.

Overall, most surgeons felt that certain forms of CBD are more effective than others, even if there was no consensus on which form was superior. Goodman et al. found that patients turn to various types of forms of CBD, but research on the effectiveness of each form is lacking [24]. Physicians and patients must consider ease of use, cost, and accessibility when deciding which forms of CBD to utilize. In addition, most surgeons believe that CBD use is overall safe, a finding well documented in the literature [25,26]. This has led to the GRAS status of Epidiolex (an oral solution), a status that may extend to multiple other products as CBD use continues to increase. However, surgeons should still be aware of and counsel patients on possible orthopaedic side effects associated with CBD use, including increased time to fracture union, wound healing, and other surgical complications [27-29]. Surgeons should also consider the effect CBD may have on habituation and mental health when considering utilizing CBD as part of their patients’ pain regimen plan [29].

Finally, the vast majority of surgeons in the current study, regardless of CBD legality in their state, believed that CBD was both affordable and easy to access for patients. While studies have documented relatively low costs for CBD ($3.68-14.71 USD, per 100mg [30]), this cost can be variable by region. Over time, this has the potential to cause a significant financial burden on patients. In addition, while surgeons do believe that CBD is accessible to their patients, they should continue to be wary of all the illegal channels that exist for CBD obtainment.

Limitations of this study are those that are inherent to a survey study. Only AOSSM members were surveyed, not allowing surgeons who are not a part of this society a chance to complete this survey. Since surveys were distributed through the AOSSM website, a true responder rate cannot be identified. There were also only a few orthopaedic surgeons who practised in CBD non-legal states that filled out this survey, a bias that could affect these results. This also precluded a comparison between responders from CBD legal and non-legal states. These two factors led to a small overall sample size. In addition, the accuracy of these answers among responders, especially by responders from CBD non-legal states, cannot be confirmed. Finally, the lack of FDA approval of CBS use for medical pain limits the ease of the completion of future studies unless these studies are FDA approved.

## Conclusions

The relative novelty of CBD inclusion in medicine has led to a lack of early education and overall experience with its use among orthopaedic sports medicine surgeons. Still, surgeons believe that CBD is a safe and effective option to control pain. Increased early education as well as continued research into the therapeutic roles of CBD can help increase its medical usage and hopefully decrease the need for opioid analgesics. As surgeons continue to gain more familiarity and trust with CBD’s medical uses over time, it has the potential to be a mainstay in orthopaedic multimodal pain regimens.

## Figures and Tables

**Figure 4:**
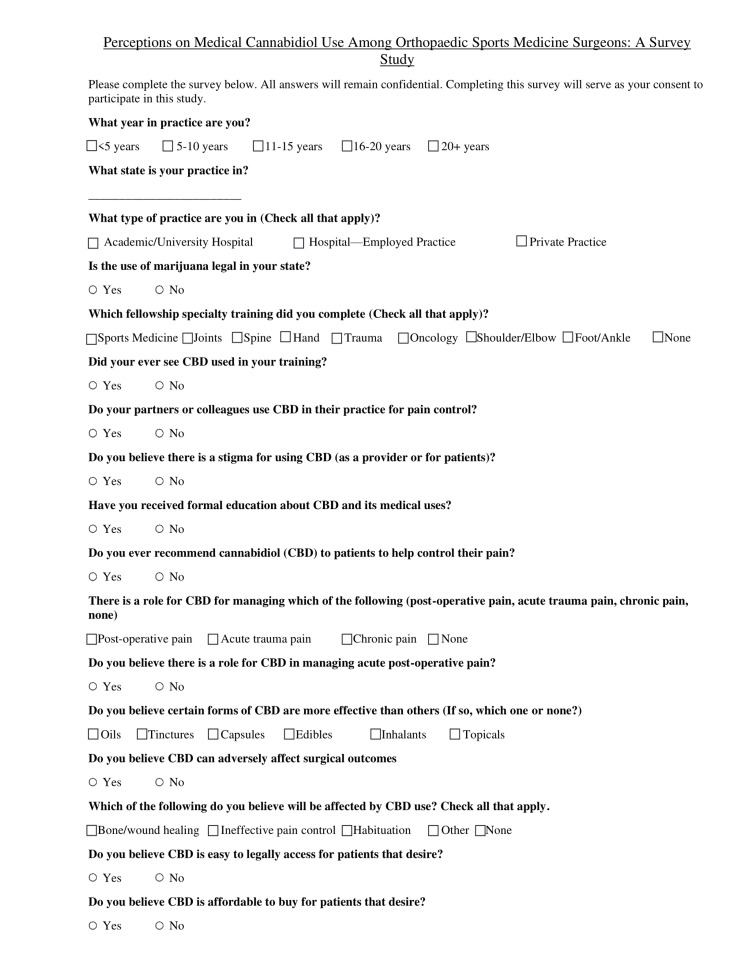
Survey for Orthopaedic Sports Medicine Surgeons
